# Patient‐Initiated Nationwide Survey on Testing for Actionable Oncogenic Drivers in Non‐Small Cell Lung Cancer in Japan

**DOI:** 10.1002/cam4.70375

**Published:** 2024-11-04

**Authors:** Satoshi Ikeda, Kazuo Hasegawa, Kenta Kachi, Akihiro Yanagisawa, Sachiko Kawakami, Shinsuke Hamasaki, Sachiko Watanabe, Aki Yoshikawa, Takayuki Takahama, Kazuhiko Nakagawa

**Affiliations:** ^1^ Department of Respiratory Medicine Kanagawa Cardiovascular and Respiratory Center Yokohama Japan; ^2^ Japan Lung Cancer Alliance Yokohama Japan; ^3^ General Incorporated Association Alliance for Lung Cancer Yokohama Japan; ^4^ Global Health Consulting Japan Tokyo Japan; ^5^ Department of Medical Oncology Kindai University Faculty of Medicine Osakasayama Japan

**Keywords:** actionable oncogenic drivers, diagnosis procedure combination, multigene testing, non‐small cell lung cancer, targeted therapy

## Abstract

**Background:**

Previous reports indicated still low implementation rates of multigene testing for advanced non‐small cell lung cancer (NSCLC) in Japan.

**Methods:**

This is a retrospective study launched at the initiative of lung cancer patients. Patients with stage IV NSCLC from January 2019 to December 2022 were investigated for testing of 8 actionable oncogenic drivers with targeted therapies available as of 2022.

**Results:**

A total of 15,719 patients were included. Between 2019 and 2022, the percentage of patients who were not tested for any actionable oncogenic drivers remained the same, ranging from 21.5% to 33.1%. However, since late 2021, the percentage of patients tested for five or more actionable oncogenic drivers has increased. Across hospital categories and regions, the number of actionable oncogenic drivers tested was similar.

**Conclusions:**

This patient‐initiated national survey in Japan reveals the recent nationwide increase in testing rates for actionable oncogenic drivers in Advanced NSCLC.

## Graphical abstract

Numbers of actionable oncogenic drivers tested were steadily increasing in Japan. Numbers of oncogenic drivers tested did not differ by hospital category or region. 25%–30% of NSCLC patients were still not tested for any actionable oncogenic drivers.

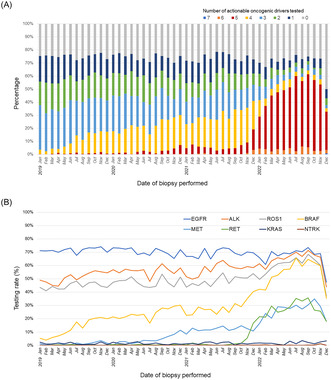

**Abbreviations**
ALK: anaplastic lymphoma kinase
BRAF: B‐Raf proto oncogene serine/threonine protein kinase
DPC: diagnosis procedure combination
EGFR: epidermal growth factor receptor
KRAS: V‐Ki‐ras2 Kirsten rat sarcoma viral oncogene homolog
MET: MNNG HOS transforming gene
NSCLC: non‐small cell lung cancer
NTRK: neurotrophic tropomyosin receptor kinase
RET: rearranged during transfection
ROS1: C‐Ros Oncogene 1
TKI: tyrosine kinase inhibitor.

## 1 Background

For non‐small cell lung cancer (NSCLC) with oncogenic driver alterations, not only conventional tyrosine kinase inhibitors (TKIs) but also a variety of targeted agents with different mechanisms of action are being developed and approved. To avoid missing the opportunity to administer these targeted therapies, medical oncologists must ensure that patients with advanced NSCLC are tested for actionable oncogenic drivers. Recently, multigene testing using next‐generation sequencing, real‐time polymerase chain reaction, or multiplex reverse transcriptome has enabled the simultaneous testing of multiple actionable oncogenic drivers. There are currently three types of multigene testing for NSCLC prior to treatment initiation which are covered by insurance in Japan; the Oncomine Dx Target Test (Thermo Fisher Scientific, Waltham, MA, USA) approved in 2019, the Amoy Dx Pan Lung Cancer PCR panel (Amoy Diagnostics Co. Ltd., Xiamen, China) approved in 2022, and the Lung Cancer Compact Panel (DNA Chip Inc., Minato‐ku, Tokyo, Japan) approved in 2023.

However, in a multicenter, retrospective “REVEAL” study conducted at 29 main institutions of West Japan Oncology Group, a leading clinical trial group in Japan, only 47.7% of advanced or recurrent NSCLC patients diagnosed between July 2020 and June 2021 underwent multigene testing, and 38.4% underwent only single‐gene testing [1]. Moreover, it is not clear whether there are differences in the rate of multigene testing or the number of actionable oncogenic drivers tested by year of diagnosis, region, or hospital type, and no reports have investigated this issue in detail.

Against these backgrounds, Kazuo Hasegawa, president of the General Incorporated Association Alliance for Lung Cancer (Hodogaya‐ku, Yokohama, Japan), a Japanese lung cancer patient advocacy group, and a lung cancer patient himself, expressed his concern that there are still many patients with actionable oncogenic drivers who do not benefit from targeted therapies because they have not been evaluated by multigene testing. Therefore, with the cooperation of medical oncologists specializing in lung cancer and Global Health Consulting Japan (Shinjuku‐ku, Tokyo, Japan), which holds the anonymously processed Diagnosis Procedure Combination (DPC) database for hospitals throughout Japan, this nationwide survey was launched. The objectives of this study were to clarify the current status of testing for actionable oncogenic drivers in Japanese NSCLC patients and to compare them by time of diagnosis, hospital category, and region.

## 2 Methods

### 2.1 Study Design and Data Source

This is a retrospective observational study using an anonymously processed data from the DPC database, a Japanese national inpatient database for acute‐care inpatients. Of the 162,563 patients from 263 hospitals with a history of hospitalization for “lung cancer” between January 2019 and December 2022, patients with (1) NSCLC, (2) Stage IV according to the 7th Edition of the TNM Staging System for Lung Cancer, and (3) transbronchial or percutaneous needle lung biopsy performed during hospitalization were included in the study (Figure S1). For these patients, we investigated the details of subsequent testing for eight oncogenic drivers, including Epidermal Growth Factor Receptor (EGFR) mutation, Anaplastic lymphoma kinase (ALK) rearrangement, C‐Ros Oncogene 1 (ROS1) rearrangement, B‐Raf Proto Oncogene Serine/Threonine Protein Kinase (BRAF) V600E mutation, MNNG HOS Transforming gene (MET) exon 14 skipping mutation, Rearranged during Transfection (RET) rearrangement, V‐Ki‐ras2 Kirsten rat sarcoma viral oncogene homolog (KRAS) G12C mutation, and Neurotrophic Tropomyosin Receptor Kinase (NTRK) fusion.

### 2.2 Ethics Approval and Consent to Participate

The information used in this analysis was from the anonymously processed DPC database for hospitals throughout Japan. This information corresponds to “Anonymously Processed Information” under Act on the Protection of Personal Information of Japan, and is therefore not subject to the “Ethical Guidelines for Medical and Health Research Involving Human Subjects” in Japan. Therefore, the approval of the ethical committee was not required. The need for obtaining individual informed consent was also waived for this study as that data were anonymized.

## 3 Results

### 3.1 Characteristics of Hospitals and Patients

A total of 15,719 patients were included in this study (Figure S1). Regarding hospital categories, “designed cancer care hospitals” by the Ministry of Health, Labour and Welfare of Japan to provide high‐quality cancer care accounted for 63.2% (129 hospitals) and included 65.4% of the patients (Table S1). Adenocarcinoma was present in 25.6%, squamous cell carcinoma in 8.3%, and 66.1% had no more detailed histological information than NSCLC.

### 3.2 Annual Change of Actionable Oncogenic Drivers Tested

**FIGURE 1:**
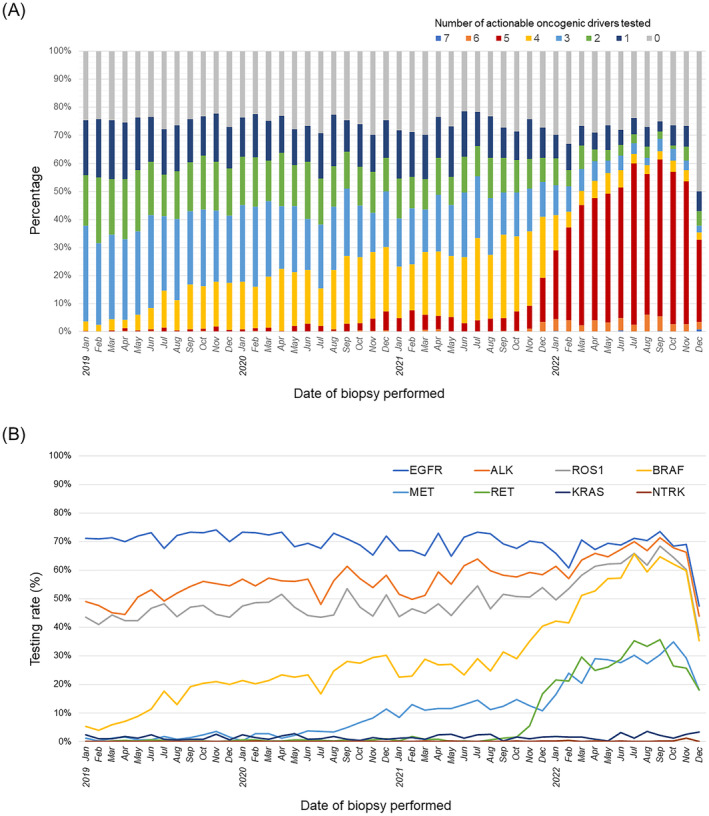
Annual change of actionable oncogenic drivers tested. Bar chart showing the annual change in the number of actionable oncogenic drivers tested (A) and line chart showing the annual change in testing rates for each oncogenic drivers (B) for patients for whom lung biopsy was performed from January 2019 to December 2022. EGFR, Epidermal Growth Factor Receptor; ALK, Anaplastic lymphoma kinase; ROS1, C‐Ros Oncogene 1; BRAF, B‐Raf Proto Oncogene Serine/Threonine Protein Kinase; MET, MNNG HOS Transforming gene; RET, Rearranged during Transfection; KRAS, V‐Ki‐ras2 Kirsten rat sarcoma viral oncogene homolog; NTRK, Neurotrophic Tropomyosin Receptor Kinase.

The annual change in the number of actionable oncogenic drivers tested is shown in Figure 1A. Between January 2019 and December 2022, the percentage of patients who were not tested for any actionable oncogenic drivers remained the same, ranging from 21.5% to 33.1%. However, since late 2021, the percentage of patients tested for five or more actionable oncogenic drivers has increased dramatically.

Subsequently, Figure 1B shows the annual change in testing rates for each oncogenic driver. While the testing rate for *EGFR* mutations remained around 70%, the testing rate for *ALK* and *ROS1* rearrangement, which was approximately 40%–50% as of January 2019, gradually increased, reaching the same level as *EGFR* mutation as of 2022. In addition, since the latter half of 2021, the testing rates for *BRAF V600E* mutation, *MET exon 14* skipping mutation, and *RET* rearrangement have been rapidly increasing.

### 3.3 Comparison of the Number of Actionable Oncogenic Drivers Tested by Hospital Category, Region in Japan, and Patient Age

**FIGURE 2:**
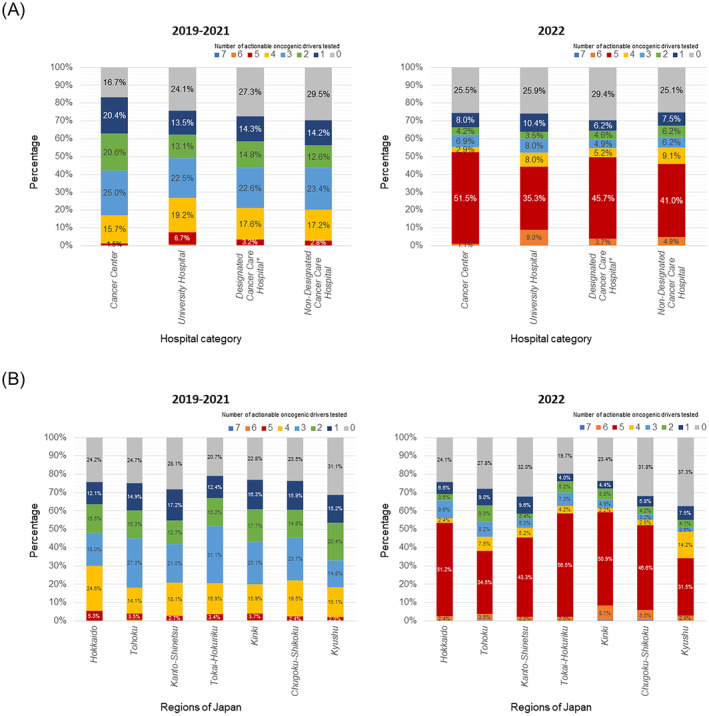
Comparison of the number of actionable oncogenic drivers tested by hospital category and region. Bar charts comparing the number of actionable oncogenic drivers tested by hospital category (A) and by region in Japan (B). Patients who underwent lung biopsy from 2019 to 2021 (left) and those who underwent lung biopsy in 2022 (right) are shown separately. *A “designed cancer care hospital” is a hospital designated by the Ministry of Health, Labour and Welfare of Japan based on the recommendation of prefectural governments to provide high quality cancer treatment.

When compared across the four hospital categories, there was no significant difference in the number of actionable oncogenic drivers tested in 2019–2021 and in 2022 (Figure 2A). When compared by region in Japan, the percentage of patients who were not tested for any actionable oncogenic driver was relatively lower in Tokai‐Hokuriku (20.7% in 2019–2021 and 19.7% in 2022). However, among patients who were tested for at least one actionable oncogenic driver in 2022, more than half of patients were tested for five or more oncogenic drivers in all regions (Figure 2B). The percentage of patients who were not tested for any actionable oncogenic drivers tended to increase with age (Figure S2).

### 3.4 Characteristics of the Patients Who Were Not Tested for any Actionable Oncogenic Drivers

When comparing the baseline characteristics of patients with no actionable oncogenic drivers tested (*N* = 4074) and those with one or more actionable oncogenic drivers tested (*N* = 11,645), the proportion of males (76.4%) and squamous cell carcinoma (17.1%) was high, and the proportion of adenocarcinoma was low (15.8%) in patients with no actionable oncogenic drivers tested (Table S2).

## 4 Discussion

This study demonstrated three important findings. First, the number of actionable oncogenic drivers tested has been steadily increasing since late 2021. Second, across hospital categories and regions of Japan, the number of actionable oncogenic drivers tested was similar. Third, there were still 25%–30% of patients who were not tested for any actionable oncogenic drivers.

In the REVEAL study of Japanese patients in July 2020 and June 2021, the rate of multigene testing was low [1]. However, the present study showed a marked increase in the number of actionable oncogenic drivers tested since late 2021. One reason may be that various targeted drugs against new oncogenic drivers have been launched one after another in Japan, including the *RET* inhibitor selpercatinib and the *KRAS G12C* inhibitor sotorasib. Another reason may be an increase in proficiency to conventional multigene testing and an increase in options for multigene testing methods. Initial concerns about the low success rate of the Oncomine Dx Target Test (Thermo Fisher Scientific, Waltham, MA, USA) [2], the first multigene testing approved in 2019, have been dispelled by recent widespread knowledge of appropriate tissue sampling and processing [3]. Moreover, the approval of the Amoy Dx Pan Lung Cancer PCR panel (Amoy Diagnostics Co. Ltd., Xiamen, China) [4] in January 2022 may also have contributed. In February 2023, the Lung Cancer Compact Panel (DNA Chip Inc., Minato‐ku, Tokyo, Japan) [5], which can be used for microtissue and cytology specimens, has also been approved, so the number of actionable oncogenic drivers tested is expected to increase even more recently.

The overall increase in the number of actionable oncogenic drivers tested, regardless of hospital category or region, indicates the high quality of medical care in Japan as a whole. In the Japanese healthcare environment, where many community hospitals as well as cancer centers and university hospitals are involved in the pharmacotherapy of lung cancer, the results of this study can provide reassurance to many patients, especially those outside of urban areas.

The fact that 25%–30% of patients are still not tested for any actionable oncogenic drivers is an issue that needs to be resolved. The high proportion of squamous cell carcinomas in patients who were not tested may be partly due to the rare detection of common oncogenic drivers such as *EGFR* and *ALK*. However, patients with new oncogenic drivers such as *MET*, *BRAF*, and *KRAS* have a higher proportion of smokers [6, 7, 8]. In that context, the frequency of *MET exon14* skipping mutation has been reported to be as high as 2%, even in squamous cell carcinoma [8]. Medical oncologists should perform multigene testing more thoroughly to avoid missing out on promising treatment options.

There are several limitations to this study. The DPC used in this study is Japan's case‐mix patient classification system, which is linked to the lump‐sum payment system for inpatients in acute care hospitals. Due to this nature, while it is a very large database, the main items are summarized in a simplified form, and detailed information on patient characteristics and treatment is often insufficient. This study cannot reveal the actual situation regarding missed testing and missed treatment opportunities for actionable oncogenic drivers in patients who underwent lung biopsy prior to the approval of multigene testing in 2019.

## 5 Conclusions

This patient‐initiated national survey in Japan reveals a recent nationwide increase in testing rates for actionable oncogenic drivers in Advanced NSCLC. Meanwhile, there remain patients who have not been adequately tested for actionable oncogenic drivers. Future updates of data like the present study will help further dissemination of multigene testing for NSCLC.

## Author Contributions

**Satoshi Ikeda:** conceptualization (equal), investigation (equal), methodology (equal), visualization (equal), writing – original draft (equal). **Kazuo Hasegawa:** conceptualization (equal), investigation (equal), methodology (equal), visualization (equal), writing – review and editing (equal). **Kenta Kachi:** investigation (equal), methodology (equal), visualization (equal), writing – review and editing (equal). **Akihiro Yanagisawa:** conceptualization (equal), methodology (equal), project administration (equal), writing – review and editing (equal). **Sachiko Kawakami:** investigation (equal), methodology (equal), writing – review and editing (equal). **Shinsuke Hamasaki:** investigation (equal), methodology (equal), writing – review and editing (equal). **Sachiko Watanabe:** data curation (equal), formal analysis (equal), methodology (equal), writing – review and editing (equal). **Aki Yoshikawa:** data curation (equal), formal analysis (equal), methodology (equal), writing – review and editing (equal). **Takayuki Takahama:** investigation (equal), methodology (equal), writing – review and editing (equal). **Kazuhiko Nakagawa:** conceptualization (equal), investigation (equal), methodology (equal), project administration (equal), supervision (equal), writing – review and editing (equal).

## Ethics Statement

The database information used in this analysis was anonymously processed by Global Health Consulting Japan from the DPC database. This information corresponds to “Anonymously Processed Information” under Act on the Protection of Personal Information of Japan, and is therefore not subject to the “Ethical Guidelines for Medical and Health Research Involving Human Subjects” in Japan. Therefore, the approval of the ethical committee was not required.

## Consent

The need for obtaining individual informed consent was waived for this study as the data were anonymized.

## Conflicts of Interest

Satoshi Ikeda received honoraria from AstraZeneca, Bristol Myers Squibb, Ono, Taiho, Chugai, Boehringer Ingelheim, Eli Lilly, Takeda, Pfizer, MSD, Amgen, and Novartis; research funding from AstraZeneca and Chugai; and took on a consulting or advisory roles for AstraZeneca, Chugai and Daiichi Sankyo. Kazuo Hasegawa, Kenta Kachi, Akihiro Yanagisawa, Sachiko Kawakami, Shinsuke Hamasaki, Sachiko Watanabe, and Aki Yoshikawa have no conflicts of interest to declare. Takayuki Takahama received honoraria from AstraZeneca, Chugai, Roche Diagnostics, and MSD; research funding from Takeda and Pfizer. Kazuhiko Nakagawa received honoraria from AstraZeneca, Ono, Chugai, Boehringer Ingelheim, Taiho, Takeda, MSD, Merck, Bayer, Nippon Kayaku, Amgen. Japan Clinical Research Operations, Taiyo Pharma, Eli Lilly, Pfizer, Novartis, Daiichi Sankyo, Bristol Myers Squibb, Janssen, Otsuka, Hisamitsu, CMIC ShiftZero, CMIC Co. Ltd., Incyte biosciences, M3, Inc., Global Health Consulting Japan, YODOSHA, Medical Mobile Communications, Life Technologies and Neo Communication; research funding from AstraZeneca, Chugai, Ono, Daiichi Sankyo, Boehringer Ingelheim, Taiho, IQVIA, EPS Corporation, Bayer, MSD, Otsuka, EPS International, PRA Health Science, Labcorp Development Japan, GlaxoSmithKline, Mochida Pharmaceutical, Japan Clinical Research Operations, Sanofi, Medical Research Support, SYNEOS HEALTH, Nippon Kayaku, Mebix, Janssen, Eli Lilly, Amgen, Novartis, SRL Diagnostics, Takeda, Eisai, Bristol‐Myers Squibb, EP‐CRSU, Pfizer, CMIC, Kobayashi Pharmaceutical, Shionogi, Astellas and Ascent Development Services; and took on a consulting or advisory roles for Eli Lilly and Ono.

## Supporting information


Data S1.

Figure S1.

Figure S2.

## Data Availability

All data generated or analyzed during this study are included in this published article.
