# Supplementary material for: Patient‐Initiated Nationwide Survey on Testing for Actionable Oncogenic Drivers in Non‐Small Cell Lung Cancer in Japan
Source: Cancer Med. 2024 Nov 4;13(21):e70375. doi: 10.1002/cam4.70375 (PMC11532810; doi:10.1002/cam4.70375)
Supplement: Supplementary file 1 — Data S1. [file CAM4-13-e70375-s003.docx]

**Table**

**Supplementary Table 1. Characteristics of Hospitals and Patients**

| **HOSPITAL CHARACTERISTICS** |  |  |  |  |  |
| --- | --- | --- | --- | --- | --- |
|  | Number of hospitals | |  | Number of patients | |
|  | (N=204) | % |  | (N=15,719) | % |
| **Category** |  |  |  |  |  |
| Cancer Center | 13 | 6.4% |  | 2,834 | 18.0% |
| University Hospital | 8 | 3.9% |  | 1,031 | 6.6% |
| Designated Cancer Care Hospital * | 129 | 63.2% |  | 10,282 | 65.4% |
| Non-Designated Cancer Care Hospital | 54 | 26.5% |  | 1,572 | 10.0% |
| **Number of beds** |  |  |  |  |  |
| 500- | 60 | 29.4% |  | 7,380 | 46.9% |
| 400-499 | 43 | 21.1% |  | 3,451 | 22.0% |
| 300-399 | 44 | 21.6% |  | 3,309 | 21.1% |
| 200-299 | 37 | 18.1% |  | 1,281 | 8.1% |
| 199 or less | 20 | 9.8% |  | 298 | 1.9% |
| **Region** |  |  |  |  |  |
| Hokkaido | 10 | 4.9% |  | 727 | 4.6% |
| Tohoku | 17 | 8.3% |  | 1,258 | 8.0% |
| Kanto-Shinetsu | 60 | 29.4% |  | 4,720 | 30.0% |
| Tokai-Hokuriku | 34 | 16.7% |  | 2,940 | 18.7% |
| Kinki | 32 | 15.7% |  | 2,753 | 17.5% |
| Chugoku-Shikoku | 21 | 10.3% |  | 1,375 | 8.7% |
| Kyushu | 30 | 14.7% |  | 1,946 | 12.4% |
|  |  |  |  |  |  |
| **PATIENT CHARACTERISTICS** |  |  |  |  |  |
|  |  |  |  | Number of patients | |
|  |  |  |  | N | ％ |
| **Age** |  |  |  |  |  |
| 39 or less |  |  |  | 112 | 0.7% |
| 40-49 |  |  |  | 481 | 3.1% |
| 50-59 |  |  |  | 1,434 | 9.1% |
| 60-69 |  |  |  | 4,091 | 26.0% |
| 70-79 |  |  |  | 7,316 | 46.5% |
| 80-89 |  |  |  | 2,204 | 14.0% |
| 90- |  |  |  | 81 | 0.5% |
| **Gender** |  |  |  |  |  |
| Male |  |  |  | 10,700 | 68.1% |
| Female |  |  |  | 5,019 | 31.9% |
| **Histology** |  |  |  |  |  |
| Adenocarcinoma |  |  |  | 4,026 | 25.6% |
| Squamous Cell Carcinoma |  |  |  | 1,301 | 8.3% |
| Non-Small Cell Lung Cancer  (No information on detailed histological type) |  |  |  | 10,392 | 66.1% |

* A "designed cancer care hospital" is a hospital designated by the Ministry of Health, Labour and Welfare of Japan based on the recommendation of prefectural governments to provide high quality cancer treatment.

**Supplementary Table 2.** **Patient characteristics according to the number of driver mutation tested.**

|  |  | No actionable oncogenic drivers tested (N=4,074) | |  | One or more actionable oncogenic drivers tested (N=11,645) | |
| --- | --- | --- | --- | --- | --- | --- |
|  |  | Number | ％ |  | Number | ％ |
| **Age** |  |  |  |  |  |  |
| 39 or less |  | 18 | 0.4% |  | 94 | 0.8% |
| 40-49 |  | 94 | 2.3% |  | 387 | 3.3% |
| 50-59 |  | 314 | 7.7% |  | 1,120 | 9.6% |
| 60-69 |  | 1,020 | 25.0% |  | 3,071 | 26.4% |
| 70-79 |  | 2,008 | 49.3% |  | 5,308 | 45.6% |
| 80-89 |  | 593 | 14.6% |  | 1,611 | 13.8% |
| 90- |  | 27 | 0.7% |  | 54 | 0.5% |
| **Gender** |  |  |  |  |  |  |
| Male |  | 3,111 | 76.4% |  | 7,589 | 65.2% |
| Female |  | 963 | 23.6% |  | 4,056 | 34.8% |
| **Histology** |  |  |  |  |  |  |
| Adenocarcinoma |  | 645 | 15.8% |  | 3,381 | 29.0% |
| Squamous Cell Carcinoma |  | 697 | 17.1% |  | 604 | 5.2% |
| Non-Small Cell Lung Cancer  (No information on detailed histological type) |  | 2,732 | 67.1% |  | 7,660 | 65.8% |

**Figure legend**

**Supplementary Figure 1. Patient disposition.**

**Supplementary Figure 2. Age comparison of the number of actionable oncogenic drivers tested.**

Bar chart comparing the number of actionable oncogenic drivers tested by patient age.
