# Supplementary figures and images for: Patient‐Initiated Nationwide Survey on Testing for Actionable Oncogenic Drivers in Non‐Small Cell Lung Cancer in Japan
Source: Cancer Med. 2024 Nov 4;13(21):e70375. doi: 10.1002/cam4.70375 (PMC11532810; doi:10.1002/cam4.70375)

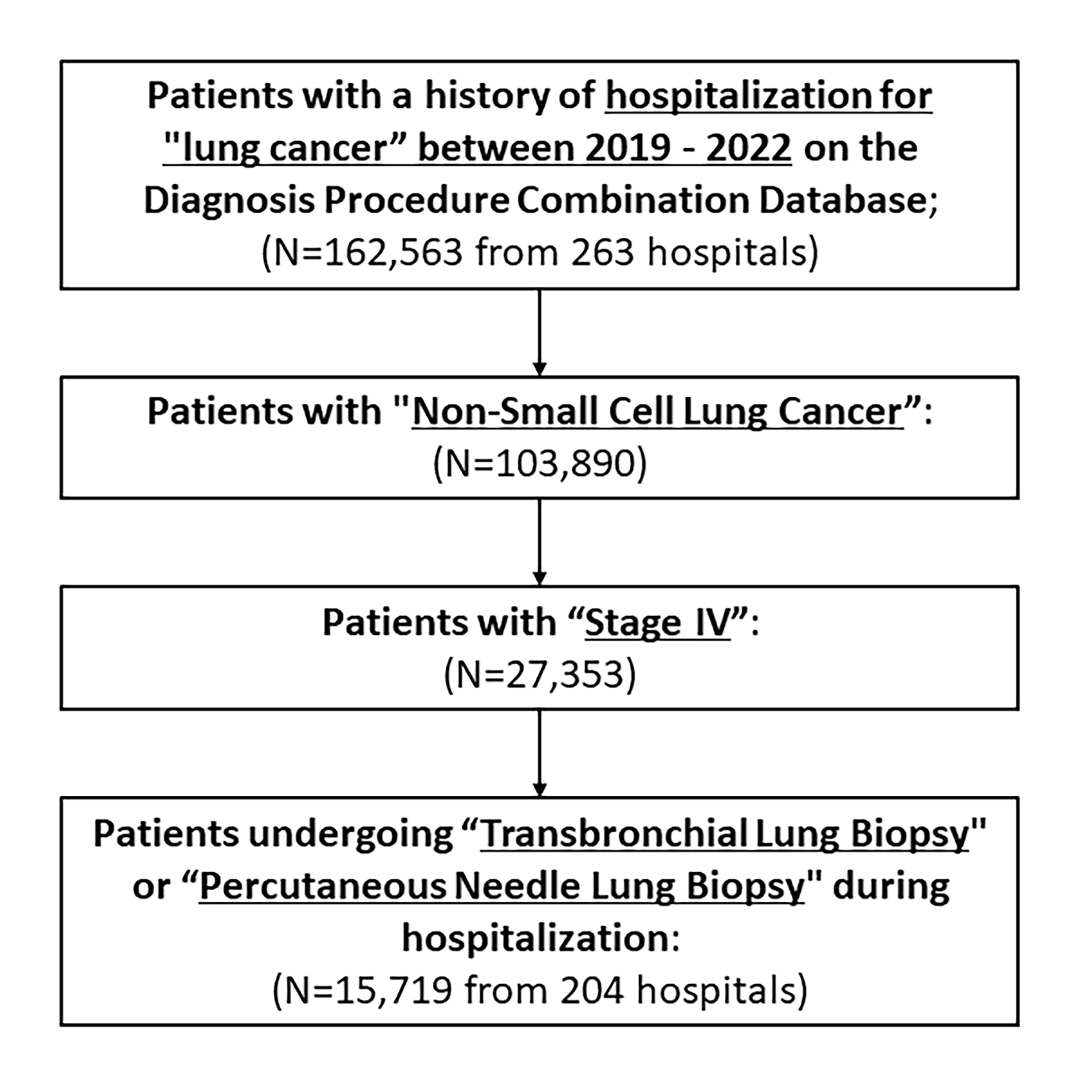

Supplement: Supplementary file 2 — Figure S1. [file CAM4-13-e70375-s002.tif]

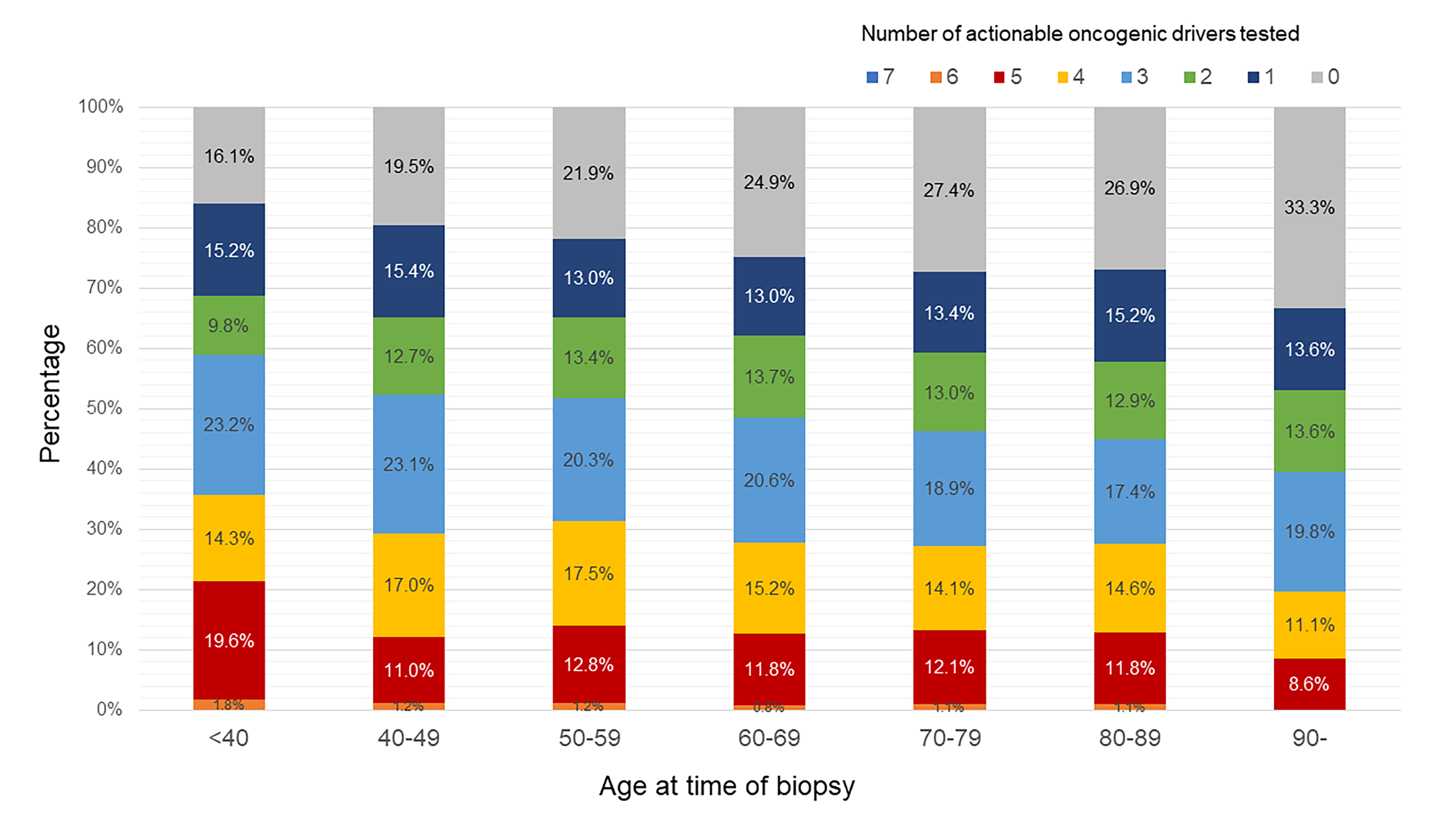

Supplement: Supplementary file 3 — Figure S2. [file CAM4-13-e70375-s001.tif]
